# Assessment of pulmonary function in COPD patients using dynamic digital radiography: A novel approach utilizing lung signal intensity changes during forced breathing

**DOI:** 10.1016/j.ejro.2024.100579

**Published:** 2024-06-27

**Authors:** Noriaki Wada, Akinori Tsunomori, Takeshi Kubo, Takuya Hino, Akinori Hata, Yoshitake Yamada, Masako Ueyama, Mizuki Nishino, Atsuko Kurosaki, Kousei Ishigami, Shoji Kudoh, Hiroto Hatabu

**Affiliations:** a Center for Pulmonary Functional Imaging, Department of Radiology, Brigham and Women’s Hospital, Harvard Medical School, 75 Francis St, Boston, MA 02115, USA; b R&D Promotion Division, Healthcare Business Headquarters, KONICA MINOLTA, INC., 2970 Ishikawa-machi, Hachioji-shi, Tokyo 192-8505, Japan; c Department of Radiology, Tenri Hospital, 200 Mishimacho, Tenri, Nara 632-8552, Japan; d Department of Clinical Radiology, Graduate School of Medical Sciences, Kyushu University, 3-1-1 Maidashi, Higashi-ku, Fukuoka, Fukuoka 812-8582, Japan; e Department of Diagnostic and Interventional Radiology, Graduate School of Medicine, Osaka University, 2-2 Yamadaoka, Suita, Osaka 565-0871, Japan; f Department of Radiology, Keio University School of Medicine, 35 Shinanomachi, Shinjuku-ku, Tokyo 160-8582, Japan; g Department of Health Care, Fukujuji Hospital, Japan Anti-Tuberculosis Association, 3-1-24 Matsuyama, Kiyose, Tokyo 204-8522, Japan; h Department of Diagnostic Radiology, Fukujuji Hospital, Japan Anti-Tuberculosis Association, 3-1-24 Matsuyama, Kiyose, Tokyo 204-8522, Japan; i Department of Respiratory Medicine, Fukujuji Hospital, Japan Anti-Tuberculosis Association, 3-1-24 Matsuyama, Kiyose, Tokyo 204-8522, Japan

**Keywords:** Dynamic digital radiography, Pulmonary function, COPD, Lung signal intensity

## Abstract

**Objectives:**

To investigate the association of lung signal intensity changes during forced breathing using dynamic digital radiography (DDR) with pulmonary function and disease severity in patients with chronic obstructive pulmonary disease (COPD).

**Methods:**

This retrospective study included 46 healthy subjects and 33 COPD patients who underwent posteroanterior chest DDR examination. We collected raw signal intensity and gray-scale image data. The lung contour was extracted on the gray-scale images using our previously developed automated lung field tracking system and calculated the average of signal intensity values within the extracted lung contour on gray-scale images. Lung signal intensity changes were quantified as SImax/SImin, representing the maximum ratio of the average signal intensity in the inspiratory phase to that in the expiratory phase. We investigated the correlation between SImax/SImin and pulmonary function parameters, and differences in SImax/SImin by disease severity.

**Results:**

SImax/SImin showed the highest correlation with VC (r_s_ = 0.54, P < 0.0001), followed by FEV_1_ (r_s_ = 0.44, P < 0.0001), both of which are key indicators of COPD pathophysiology. In a multivariate linear regression analysis adjusted for confounding factors, SImax/SImin was significantly lower in the severe COPD group compared to the normal group (P = 0.0004) and mild COPD group (P=0.0022), suggesting its potential usefulness in assessing COPD severity.

**Conclusion:**

This study suggests that the signal intensity changes of lung fields during forced breathing using DDR reflect the pathophysiology of COPD and can be a useful index in assessing pulmonary function in COPD patients, potentially improving COPD diagnosis and management.

## Highlights

• This is the first report to examine signal intensity change ratio (SImax/SImin) of the entire lungs during forced breathing in COPD patients using DDR.
• Entire lung signal intensity ratio showed moderate correlation with VC and FEV_1_.
• In a multilinear regression analysis, the severe COPD group had a lower entire lung signal intensity ratio compared to the normal group and mild COPD group.
• Lung signal intensity changes using DDR reflect the pathophysiology of COPD and can be a useful index in assessing pulmonary function in COPD patients.

## 1 Introduction

Chronic obstructive pulmonary disease (COPD), mainly caused by exposure to tobacco smoke, is the third leading cause of death worldwide with 3.23 million deaths in 2019 [1], [2]. COPD causes irreversible airflow restriction due to small airway changes and lung damage, leading to lung hyperinflation at rest and during exercise. [3], [4]. The pathophysiology is reflected in spirometry measurements. Specifically, airflow obstruction leads to a progressive decrease of forced expiratory volume in one second (FEV_1_) [3], whereas disease progression causes an increase in residual volume that exceeds an increase in total lung capacity, resulting in a decrease in vital capacity (VC) [4], [5]. Reduced VC leads to dyspnea and frequently restricts exercise [4], [5], [6]. Disease severity in COPD is evaluated based on percent predicted forced expiratory volume in one second (%FEV_1_) and the presence of symptoms or risk of exacerbations [7].

Dynamic digital radiography (DDR) is a novel functional imaging technique that utilizes sequential images obtained by a pulsed X-ray generator and a flat panel detector (FPD) with a large field of view and can be used in routine clinical practice [8], [9], [10]. DDR can not only quantitatively assess pulmonary ventilation and perfusion but also offer a non-invasive and comprehensive evaluation of pulmonary function, potentially impacting clinical practice significantly [8], [9], [10], [11], [12], [13], [14], [15], [16], [17], [18], [19], [20], [21], [22], [23], [24], [25]. Previous studies have focused on the dynamic changes in X-ray translucency of the lung fields during respiration, which mainly reflect the changes of air volume inside the alveoli. These studies have demonstrated that the quantitative measurements of lung density changes were useful in assessing pulmonary function [21], [22], [23]. However, limited research has examined the utility in assessing pulmonary function in COPD patients [23]. Recently, we developed a fully automated software that continuously contours and tracks lung fields during the examination [18], facilitating the measurement of average signal intensity changes of entire lung fields on DDR images during respiration.

Spirometry, the gold standard for assessing pulmonary function in COPD patients, has the limitation of being relatively insensitive to the underlying lung morphological changes in the early stages of COPD [26]. DDR may provide additional insights into the underlying pathophysiology of COPD. We hypothesized that the signal intensity changes of lung fields during forced breathing using DDR would reflect the underlying pathophysiology of COPD and be associated with the worsening pulmonary function in COPD patients. The aim of this study is to investigate the correlations between the signal intensity changes of lung fields during forced breathing obtained by DDR with our previously developed automated lung field tracking system and the pulmonary function test (PFT) parameters in a cohort of COPD patients and normal controls, and to compare the differences in the signal intensity change by the severity of COPD.

## 2 Materials and methods

### 2.1 Population selection

This retrospective study was approved by the local institutional review board and was performed in accordance with the principles of the Declaration of Helsinki. Written informed consent was obtained from all the participants. COPD patients were recruited from June 2009 to August 2011, and healthy volunteers were recruited from May 2013 to February 2014. The inclusion criteria were the same as in previous studies [14], [16]. The common inclusion criteria for both groups were: (1) ≥ 20-year-old adults with informed consent; (2) scheduled for conventional chest radiograph; (3) ability to follow instructions for forced breathing; (4) no status of pregnancy, potential pregnancy, or lactation. The additional inclusion criteria for COPD patients were: (5) clinical diagnosis of pure COPD based on clinical course, symptoms, chest computed tomography scans, and PFTs with post-bronchodilator inhalation; (6) current or former smokers; (7) no evidence of other respiratory disease. All the COPD subjects were classified into four grades according to the global initiative for chronic obstructive lung disease (GOLD) criteria [7]. In this study, GOLD 1 and 2 (i.e., %FEV_1_ ≥ 50) and GOLD 3 and 4 (i.e., %FEV_1_ < 50) were defined as mild COPD and severe COPD, respectively. In contrast, the additional inclusion criteria for healthy volunteers were: (5) PFT results within normal limits, namely percent predicted vital capacity (%VC) > 80 % and the ratio of FEV_1_ to forced VC (FEV_1_%) > 70 %; (6) never smokers; (7) no past medical history of respiratory diseases. The exclusion criteria were patients with incomplete datasets of DDR. These selection timelines and criteria were established to develop DDR-related technologies and explore their clinical relevance.

### 2.2 Image protocol of dynamic digital radiography

DDR of the posteroanterior (PA) view was performed in the standing position using a prototype X-ray system (Konica Minolta Inc., Tokyo, Japan) composed of an FPD (PaxScan 4030CB, Varian Medical Systems Inc., Salt Lake City, UT, USA) and a pulsed X-ray generator (DHF-155HII with Cineradiography option, Hitachi Medical Corporation, Tokyo, Japan). Healthy volunteers took several tidal breaths followed by one forced breath, while COPD patients took tidal breaths and a forced breath separately. Imaging parameters for DDR were almost the same as the previous reports [16], [18]: tube voltage, 100 kV; tube current, 50 mA; pulse duration of pulsed X-ray, 1.6 ms; source-to-image distance, 2 m; additional filter, 0.5 mm Al plus 0.1 mm Cu. The additional filter was used to remove soft X-rays. The exposure time was approximately 10–15 s. The pixel size was 388 × 388 μm, the matrix size was 1024 × 768, and the overall image area was 40 × 30 cm. Dynamic image data at 7.5 frames/second (COPD patients) or 15 frames/second (healthy volunteers) were captured with synchronized pulsed X-rays which prevented excessive radiation exposure to the subject. The entrance surface dose was approximately 0.3–1.0 mGy.

### 2.3 Image data collection and analysis

Two sets of data were collected from a DDR examination: signal intensity data and gray-scale image data. Signal intensity value is unitless digitized data generated by sensors inside the flat panel detector. The signal intensity is raw data, i.e., uncorrected output value from a flat panel. The signal intensity is proportional to the incident exposure of the X-ray detector. The images with 65,536 (16-bit) gray-scale levels are generated from raw data through data correction and image processing for quality improvement. These are DDR images used in daily clinical practice. In this study, we focused on raw data before any image processing to measure signal intensity values. The core principle of DDR is the analysis and imaging of dynamic changes on the basis of X-ray translucency [9]. In this context, we considered that the raw data more directly reflects respiratory changes in the lungs compared to processed images for diagnostic purposes. The raw data does not exhibit collinearity.

Bilateral lung contours were drawn on the DDR images. The contour extraction was performed using prototype software with standard settings (Konica Minolta Inc., Tokyo, Japan) installed in a standalone workstation (Operating system: Windows 7 Professional 64-bit Service Pack 1; Microsoft, Redmond WA; CPU: Intel® CoreTM i5–6500, 3.20 GHz; random access memory, 16 GB). Lung contour detection was fully automated, and the accuracy and reproducibility of contour extraction in a previous study [18]. The average signal intensity values within the corresponding lung contour on gray-scale images were calculated on all image frames.

A time-intensity curve was drawn, and the maximum average signal intensity of lung fields in inspiratory phase (SImax) and the minimum average signal intensity of lung fields in expiratory phase (SImin) during forced breathing were determined. The entire lung signal intensity ratio was defined as SImax divided by SImin (SImax/SImin), which represents the maximum rate of change in signal intensity in the lung fields during forced breathing.

### 2.4 Pulmonary function tests

All participants underwent PFTs on the same day of DDR. A pulmonary function instrument with computer processing (DISCOM-21 FX, Chest MI Co, Tokyo, Japan) was used for PFTs. The following parameters were obtained according to the guideline of American Thoracic Society [27]: tidal volume (TV), VC, %VC, FEV_1_, FEV_1_%, and %FEV_1_.

### 2.5 Statistical analysis

Demographics and PFT parameters were compared among the three groups, including normal, mild COPD mild, and severe COPD, using one-way analysis for continuous variables (age, height, weight, body mass index [BMI], TV, VC, %VC, FEV_1_, FEV_1_%, and %FEV_1_) and Fisher’s exact test for categorical variables (gender). Multiple comparisons were adjusted by using the Bonferroni method. The correlations between SImax/SImin and PFT parameters were analyzed using the Spearman’s rank correlation test. A correlation was considered strong if the absolute value of the correlation coefficient (r_s_) was > 0.7, moderate if the r_s_ was ≤ 0.7 to > 0.4, weak if the r_s_ was ≤ 0.4 to > 0.2, and absent if the r_s_ was ≤ 0.2. The difference in SImax/SImin among the three groups was analyzed using the Wilcoxon rank-sum test. The associations between SImax/SImin and COPD severity were assessed with a multivariate linear regression analysis after adjusting for potential confounding factors. PFT parameters were excluded from confounders to avoid multicollinearity since COPD severity is derived from PFT parameters. All statistical analyses were performed using JMP Pro16.0.0 (SAS Institute Inc., Cary, NC, USA). A two-sided P value less than 0.05 was considered significant.

## 3 Results

### 3.1 Inclusion/exclusion criteria

Thirty-seven COPD patients and 47 healthy subjects met the inclusion criteria. Four COPD patients and one healthy subject were excluded due to the incomplete dataset. Finally, a total of 33 COPD and 46 healthy subjects were analyzed in this study. According to the results of PFT, 15 of the 33 COPD patients were classified as mild COPD and 18 as severe COPD, and the normal control included 46 healthy subjects.

### 3.2 Demographic and PFT data

The demographic and PFT data of the participants are summarized in Table 1. Among the three groups, there were significant differences in age (P < 0.0001), sex (P = 0.0003), and the PFT parameters (TV, P = 0.0054; VC, P = 0.0007; and the others, P < 0.0001), and there were no significant differences in height, weight, and BMI. Multiple comparisons showed significant differences in age and sex between the normal and the mild COPD groups and between the normal and the severe COPD groups; in TV between the normal and the mild COPD groups; in VC between the normal and the severe COPD groups; in %VC between the mild and the severe COPD groups and between the normal and the severe COPD groups; and in FEV_1_, FEV_1_%, and %FEV_1_ between the three groups.

**Table 1:** Demographic data and pulmonary function tests among Normal, mild COPD, and severe COPD groups.

	**Normal**	**Mild COPD**	**Severe COPD**	**P value**
	**(n=46)**	**(n=15)**	**(n=18)**	
**Demographic data**				
**Age (years)**	54.8 ± 9.9	74.3 ± 10.0^a^	69.1 ± 9.1^c^	<0.0001
	[36–72]	[48–85]	[54–85]	
**Sex, Female/Male**	26 / 20	2 / 13^a^	2 / 16^c^	0.0003
**Height (cm)**	162.3 ± 9.4	163.6 ± 5.6	163.8 ± 6.8	0.75
	[146.1–183.7]	[150.0–171.0]	[150.0–176.0]	
**Weight (kg)**	59.2 ± 10.3	58.1 ± 6.2	56.1 ± 14.1	0.59
	[37.0–78.0]	[46.0–67.0]	[42.0–94.0]	
**BMI (kg/m**^**2**^**)**	22.4 ± 2.9	21.8 ± 2.3	20.8 ± 4.5	0.23
	[15.1–31.1]	[16.3–24.2]	[16.5–34.5]	
**Smoking history**				
**Current or former**	0	15	18	
**Never**	46	0	0	
**GOLD, 1/2/3/4**	0 / 0 / 0 / 0	4 / 11 / 0 / 0	0 / 0 / 14 / 4	
**Pulmonary function**				
**TV (L)**	0.76 ± 0.35	1.12 ± 0.48^a^	0.88 ± 0.28	0.0054
	[0.34–1.76]	[0.39–2.3]	[0.54–1.67]	
**VC (L)**	3.35 ± 0.87	3.14 ± 0.58	2.47 ± 0.71^c^	0.0007
	[2.11–5.70]	[2.35–4.34]	[1.49–4.03]	
**%VC (%)**	110.4 ± 14.6	103.6 ± 17.9	77.8 ± 18.5^b^^,^^c^	<0.0001
	[92.1–159.6]	[78.2–140.0]	[44.6–111.3]	
**FEV**_**1**_**(L)**	2.72 ± 0.74	1.72 ± 0.33^a^	0.98 ± 0.29^b^^,^^c^	<0.0001
	[1.58–4.72]	[1.08–2.41]	[0.52–1.60]	
**FEV**_**1**_**% (%)**	82.4 ± 6.2	57.5 ± 6.4^a^	41.6 ± 4.4^b^^,^^c^	<0.0001
	[70.5–97.0]	[45.8–68.9]	[34.6–48.3]	
**%FEV**_**1**_**(%)**	107.2 ± 14.9	69.5 ± 12.9^a^	36.0 ± 8.8^b^^,^^c^	<0.0001
	[80.6–163.9]	[52.4–97.9]	[16.3–47.1]	

BMI, body mass index; GOLD, global initiative for chronic obstructive lung disease; TV, tidal volume; VC, vital capacity; %VC, percent vital capacity; FEV_1_, forced expiratory volume in one second; FEV1 %, forced expiratory volume percent in one second divided by forced vital capacity; %FEV_1_, percent predicted FEV_1_.

Data are presented as mean ± standard deviation [range] or number.

a The difference between normal and mild COPD groups was statistically significant (Bonferroni-adjusted P < 0.05).

b The difference between mild and severe COPD groups was statistically significant (Bonferroni-adjusted P < 0.05).

c The difference between normal and severe COPD groups was statistically significant (Bonferroni-adjusted P < 0.05).

### 3.3 Association between signal intensity change and PFT parameters

**Fig. 1:**
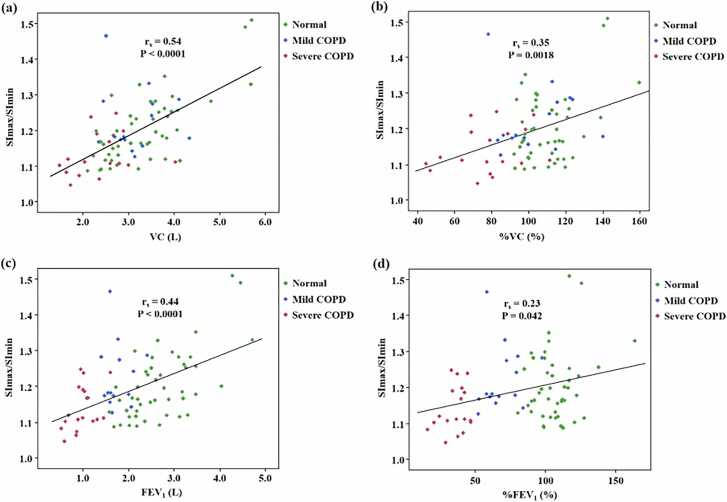
Correlation of pulmonary function parameters and SImax/SImin. The scatter plots of (a) VC, (b) %VC, (c) FEV1, and (d) %FEV1 versus SImax/SImin showed weak to moderate correlation by Spearman’s rank correlation analysis. VC, vital capacity; %VC, percent vital capacity; FEV1, forced expiratory volume in one second; %FEV1, percent predicted FEV1.

Table 2 provides the Spearman’s correlation coefficients between SImax/SImin of the bilateral lungs and PFT parameters. VC and FEV_1_ showed moderate correlations with SImax/SImin (r_s_ = 0.54 [95 % CI: 0.36, 0.68], P < 0.0001 and r_s_ = 0.44 [95 % CI: 0.25, 0.61], P < 0.0001, respectively), and %VC and %FEV_1_ showed weak correlations with SImax/SImin (r_s_ = 0.35 [95 % CI: 0.14, 0.53], P = 0.0018 and r_s_ = 0.23 [95 % CI: 0.01, 0.43], P = 0.042, respectively). There was no significant correlation between TV and FEV_1_%, and the SImax/SImin. The scatter plots of SImax/SImin in bilateral lungs and PFT parameters that showed significant correlation are presented in Fig. 1. Table S1 provides the Spearman’s correlation coefficients between the SImax/SImin of the left and right lungs and the PFT parameters. VC and FEV_1_ showed significant correlations with signal intensity changes in both the left (r_s_ = 0.56 [95 % CI: 0.39, 0.70], P < 0.0001 and r_s_ = 0.44 [95 % CI: 0.24, 0.60], P < 0.0001, respectively) and the right lungs (r_s_ = 0.44 [95 % CI: 0.25, 0.61], P < 0.0001 and r_s_ = 0.37 [95 % CI: 0.16, 0.55], P = 0.0008, respectively).

**Table 2:** Spearman’s correlation coefficients of signal intensity changes (SImax/SImin) with pulmonary function.

**Pulmonary function**	**r**_**s**_**(95 % CI)**	**P value**
**TV (L)**	0.19 (-0.04, 0.39)	0.10
**VC (L)**	0.54 (0.36, 0.68)	<0.0001
**%VC (%)**	0.35 (0.14, 0.53)	0.0018
**FEV**_**1**_**(L)**	0.44 (0.25, 0.61)	<0.0001
**FEV**_**1**_**% (%)**	0.21 (-0.01, 0.42)	0.059
**%FEV**_**1**_**(%)**	0.23 (0.01, 0.43)	0.042

TV, tidal volume; VC, vital capacity; %VC, percent vital capacity; FEV_1_, forced expiratory volume in one second; FEV_1_%, forced expiratory volume percent in one second divided by forced vital capacity; %FEV_1_, percent predicted FEV_1_, CI, confidence interval.

### 3.4 Association between signal intensity change and COPD severity

**Fig. 2:**
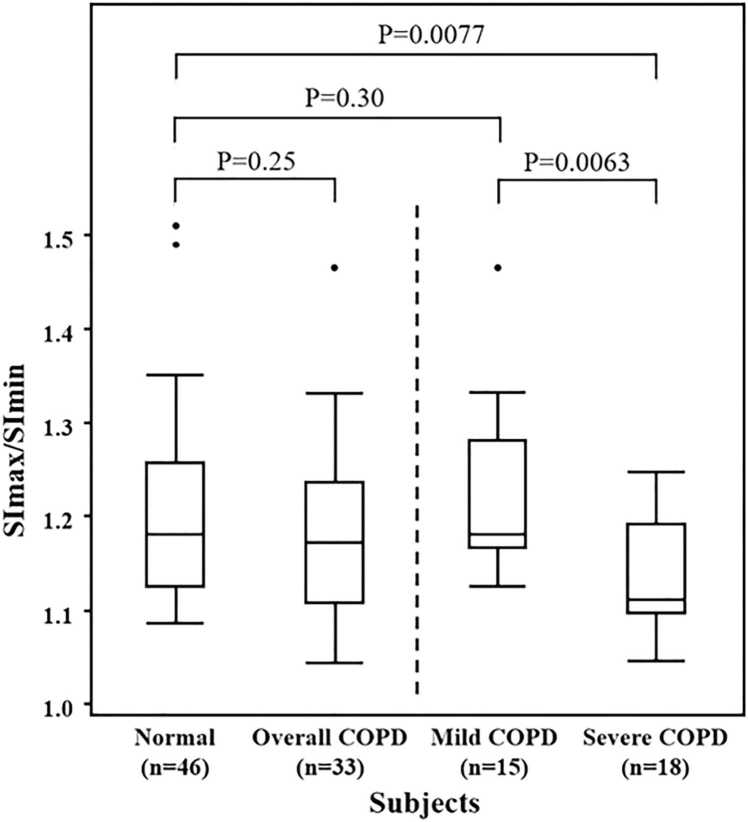
Comparison of signal intensity changes (SImax/SImin) among normal and mild/severe COPD groups.

**Fig. 3:**
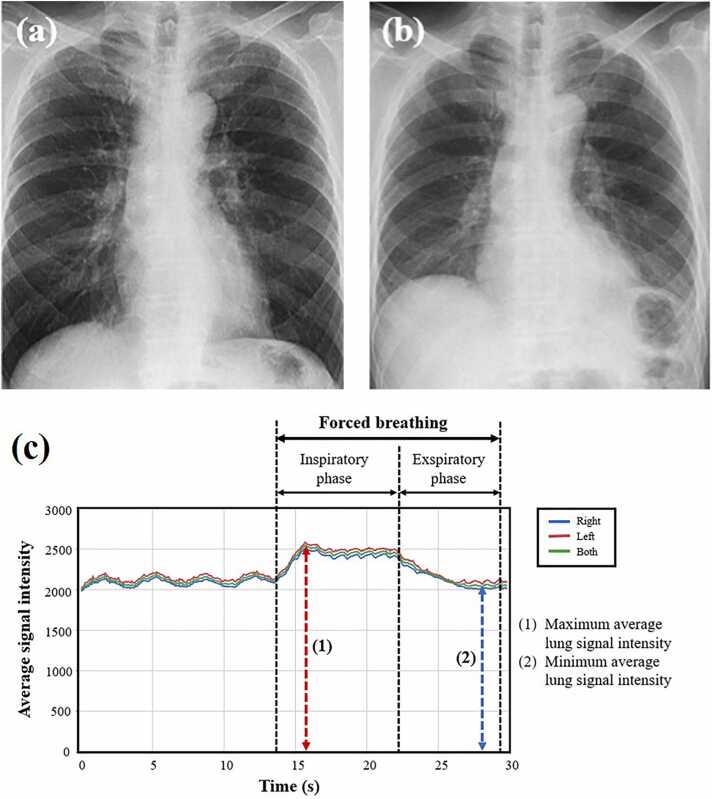
Representative chest radiographs and graphs of lung signal intensity obtained using DDR for the normal group. The subject is a 67-year-old male with VC of 3.95 L and FEV_1_ of 3.26 L. (a) Radiograph during forced inspiration when the average signal intensity reached its maximum (SImax = 2537.5). (b) Radiograph during forced expiration when the average signal intensity reached its minimum (SImin = 2026.2). (c) Curve graphs depict the temporal change in the signal intensity of entire lung and each individual lung, tracked automatically during respiration, including forced breathing. The signal intensity change (SImax/SImin) was calculated as 1.25. DDR, dynamic digital radiography; VC, vital capacity; FEV_1_, forced expiratory volume in one second.

**Fig. 4:**
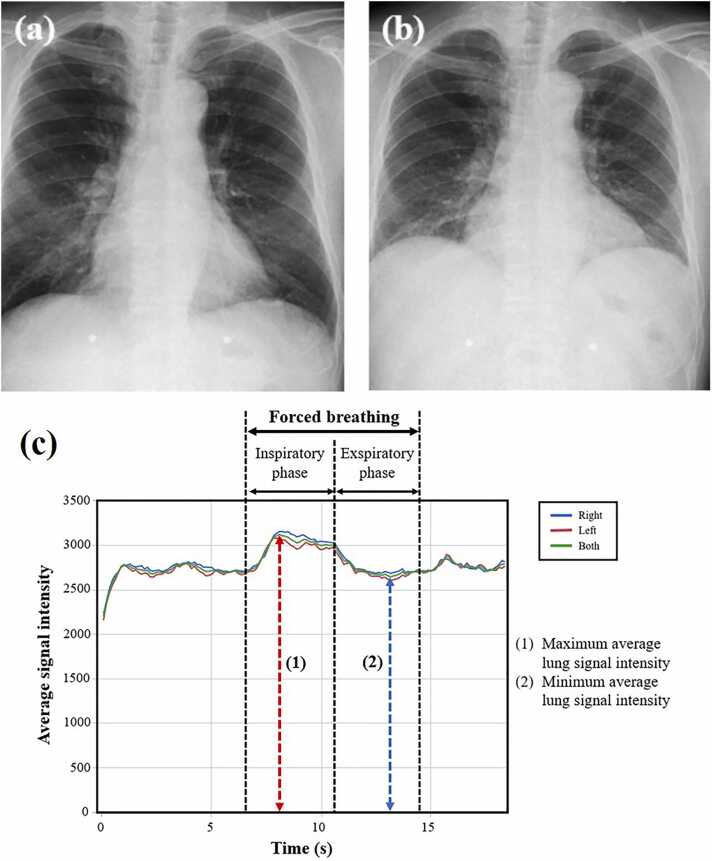
Representative chest radiographs and graphs of lung signal intensity obtained using DDR for the mild COPD group. The subject is a 75-year-old male with VC of 2.70 L and FEV_1_ of 1.61 L. (a) Radiograph during forced inspiration when the average signal intensity reached its maximum (SImax = 3113.7). (b) Radiograph during forced expiration when the average signal intensity reached its minimum (SImin = 2635.0). (c) Curve graphs depict the temporal change in the signal intensity of entire lung and each individual lung, tracked automatically during respiration, including forced breathing. The signal intensity change (SImax/SImin) was calculated as 1.18. DDR, dynamic digital radiography; VC, vital capacity; FEV_1_, forced expiratory volume in one second.

**Fig. 5:**
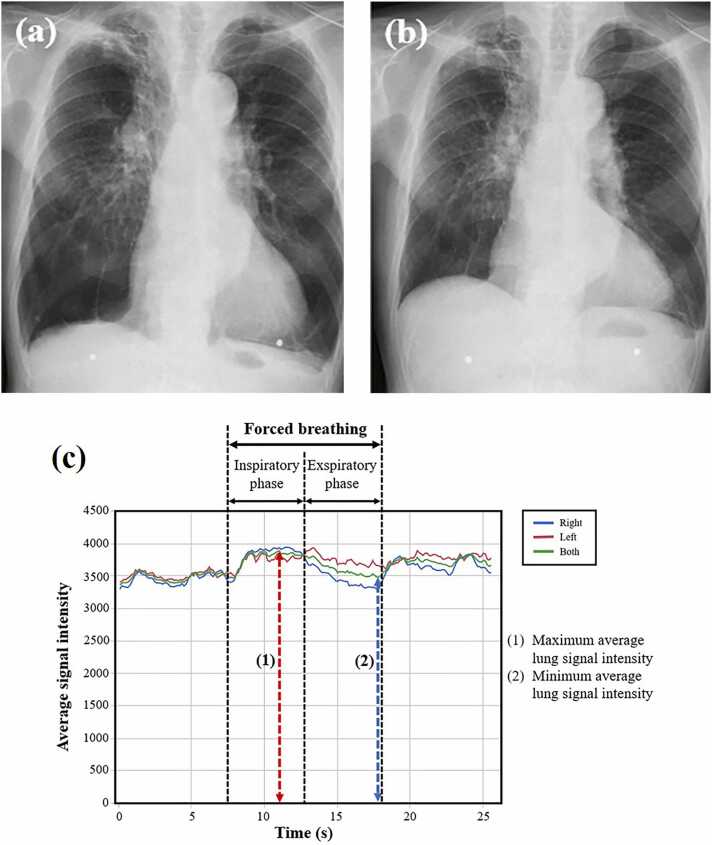
Representative chest radiographs and graphs of lung signal intensity obtained using DDR for the severe COPD group. The subject is a 77-year-old male with VC of 1.68 L and FEV_1_ of 0.68 L. (a) Radiograph during forced inspiration when the average signal intensity reached its maximum (SImax = 3879.3). (b) Radiograph during forced expiration when the average signal intensity reached its minimum (SImin = 3466.5). (c) Curve graphs depict the temporal change in the signal intensity of entire lung and each individual lung, tracked automatically during respiration, including forced breathing. The signal intensity change (SImax/SImin) was calculated as 1.12. DDR, dynamic digital radiography; VC, vital capacity; FEV_1_, forced expiratory volume in one second.

Fig. 2 shows the comparison of SImax/SImin among the normal, overall COPD, mild COPD, and severe COPD groups: the median SImax/SImin was 1.18 (interquartile range [IQR], 1.13–1.26), 1.17 (IQR, 1.11–1.24), 1.18 (IQR, 1.17–1.28), and 1.11 (IQR, 1.10–1.19), respectively. The severe COPD group had a lower SImax/SImin than the normal group (P = 0.0077) and mild COPD group (P = 0.0063). There was no significant difference in the SImax/SImin between the overall COPD and the normal groups and between the mild COPD and the normal groups. Representative cases of the normal, mild COPD, and severe COPD groups are shown in Fig. 3, Fig. 4, Fig. 5.

A multivariable linear regression analysis of SImax/SImin on the COPD severity, adjusted for age, sex, and height, was performed (Table 3). The severe COPD group had significantly lower SImax/SImin than the normal group (P = 0.0004) and the mild COPD group (P = 0.0022). However, there was no significant difference in the SImax/SImin between the mild and the normal groups. Female gender independently showed a negative association with the SImax/SImin (P = 0.028). Additionally, a multivariable linear regression analysis of SImax/SImin on the presence of COPD, adjusted for age, sex, and height, was performed (Table S2). The overall COPD group had a lower SImax/SImin than the normal group (P = 0.014). In this analysis, female gender was not significantly associated with SImax/SImin.

**Table 3:** Multivariate linear regression analysis of signal intensity changes (SImax/SImin) on COPD severity and confounding factors.

	**Estimate**	**Standard error**	**t value**	**P value**
COPD severity				
Mild	-0.005	0.015	-0.36	0.72
Severe	-0.049	0.013	-3.69	0.0004
(Ref: Normal)				
Severe	-0.044	0.014	-3.17	0.0022
(Ref: Mild)				
Confounding factors				
Age	-2.3e-5	0.001	-0.02	0.98
Sex, Female	-0.033	0.015	-2.25	0.028
(Ref: Male)				
Height	0.003	0.002	1.60	0.11

Ref, reference.

## 4 Discussion

In this study, we focused on SImax/SImin, which represents the maximum ratio of the average signal intensity in the inspiratory phase to that in the expiratory phase during forced breathing on the lung fields of DDR images. Our findings revealed that SImax/SImin had moderate correlations with VC and FEV_1_ and weak correlations with %VC and %FEV_1_. The multivariate linear regression analysis revealed significant differences in the SImax/SImin between the severe COPD and the normal groups, as well as between the severe COPD and the mild groups. While multiple studies to date have demonstrated the utility of quantitative DDR markers in assessing pulmonary function in COPD patients [10], [12], [14], [16], [17], [18], [20], [23], this is the first report to focus on the signal intensity changes of the entire lung fields on DDR images in COPD patients and investigate the correlation with PFT parameters and the differences by disease severity.

Recently, Hino et al. [18] evaluated the association between projected lung area (PLA) in tidal/forced breathing and pulmonary function in COPD patients using DDR and reported the usefulness of DDR as an alternative to spirometry. In the study, the difference of PLA between end-inspiration and end-expiration (ΔPLA) in forced breathing, the same condition as in our study, showed moderate correlation with VC (r_s_ = 0.58) [18], while the correlation between the SImax/SImin and VC (r_s_ = 0.54) in our study was almost equivalent, although it did not reach the result in the previous study. On the other hand, Hino et al. reported that ΔPLA in forced breathing showed weak correlation with FEV_1_ (r_s_ = 0.33) [18], while the SImax/SImin in our study provided a stronger correlation with FEV_1_ (r_s_ = 0.44) than the result in the previous study. Hence, our study demonstrated that DDR can simultaneously assess both VC and FEV_1_, key indicators of COPD pathophysiology, through the signal intensity changes in bilateral lung fields during forced breathing and provided new evidence regarding the utility of DDR in assessing pulmonary function in COPD patients.

Previous studies have reported that in univariate analysis, DDR markers, including PLA [18], diaphragmatic excursion [14], and craniocaudal gradients of the maximum pixel value change rate [23], tended to be significantly different in the severe COPD group compared to the normal and the mild COPD groups. In the statistical analysis of this study, we introduced multivariate linear regression analysis, which confirmed the robust results that the severe COPD group had significantly lower SImax/Simin than the other groups. The results may be because the SImax/SImin in the severe COPD group was more strongly influenced by the decline in FEV_1_ and VC. Importantly, our findings suggest that the signal intensity changes can identify the severe COPD group as effectively as the previously reported DDR markers. In contrast, the SImax/SImin in the mild COPD group was not significantly different from that in the normal group, which may be due to the heterogeneous pathophysiological characteristic of COPD [2], [3]. This inference may be supported by our PFT results that FEV_1_ showed significant differences among the three groups, while VC showed a significant difference only between the severe COPD and the normal groups. Additionally, the SImax/SImin showed significant differences in the multivariate analysis between the overall COPD group and the normal group, indicating the ability to detect physiologic decrements in COPD patients. The results of our study suggest the utility of signal intensity change using DDR as a modality for diagnosing and assessing the severity of COPD.

All previous studies have used the pixel value instead of the signal intensity to measure the changes in X-ray translucency in the lung fields which are primarily related to the changes in air volume in the alveoli [8], [21], [22], [23], [28], [29]. In general, the pixel value of the gray-scale images is inversely proportional to the logarithm of the signal intensity [23]. In animal experiments using pigs, the changes in pixel value showed a good correlation with the inspired air volume [28]. In a small clinical study involving various respiratory diseases, including emphysema, the changes in pixel value during forced breathing were shown to correlate significantly with the VC [29]. Since both the pixel value-based and the signal intensity-based methods share the basic concept, it is not surprising that these previous results are consistent with the findings of the present study.

Of note, these methods represent a novel approach to pulmonary function imaging in COPD patients in that they can detect physiological information more specific to structures within the lungs, and are different from the methods using respiratory kinetics of the diaphragm and the thorax on DDR images discussed in previous studies [10], [12], [14], [16], [17], [18], [20]. The unique approach-derived indices, particularly in the evaluation of localized areas of the lung, have shown to correlate with ventilation metrics from nuclear medicine imaging [21], [22] and could detect ventilation abnormalities such as air trapping and airflow restriction [28]. Thus, the method of our study could be applied to assess pulmonary function in various selected regions of the lung (e.g., upper, lower, central, or peripheral zones) in COPD patients, which may provide additional insights into the assessment of COPD pathophysiology and contribute to the improved diagnosis and management of COPD, when combined with spirometry.

In this study, we used the DDR prototypes with automated lung field tracking system reported in our previous work [18]. The automatically tracked PLA showed strong correlations with the manually contoured PLA (Pearson’s correlation coefficient = 0.96, p < 0.001) [18]. This method plays an important role in overcoming the barriers to its clinical implementation, including the need for trained experts and the time-consuming process of manual contouring. It should be noted that the improved versions of the DDR system and the analysis software are currently commercially available for routine clinical use, and their reliability and reproducibility will need to be evaluated additionally.

There are several limitations to this study. First, this was a retrospective single-center investigation with a small number of patients. Most DDR studies to date have faced similar limitations due to the emerging technology [25]. We used the same cohort employed in our previous studies demonstrating the utility of various quantitative DDR markers in COPD patients [14], [16], [18]. Although we did not perform a formal sample size calculation, the consistency of the results within these studies may support the validity of our cohort; however, the generalizability and robustness of our findings are limited. Second, there are technical limitations. The automated tracking system we used can extract lung fields on DDR images. It should be noted, however, that the extracted lungs do not include the lungs overlapping the diaphragm and heart because DDR, like conventional chest radiography [30], is a two-dimensional projection of a three-dimensional volume. This exclusion can lead to incomplete assessment of pulmonary function. Furthermore, the signal intensity measured in this study was based on one cycle of forced breathing in each participant, which could introduce variability. The expiratory effort of each participant was judged to be adequate, but evaluation with multi-cycle forced breathing or repeated examinations of each participant would be desirable to minimize potential effort-by-effort variability and to capture complexity of pulmonary function in COPD patients. Third, there are statistical limitations. Correlations observed with SImax/SImin do not imply causation and may be influenced by patient characteristics, comorbidities, and disease heterogeneity. Multiple regression analysis, despite its usefulness, cannot establish causality and may be affected by unaccounted confounders. Larger prospective studies with standardized methodologies are needed to evaluate the reproducibility of our findings and address these limitations regarding technique and result interpretation.

In conclusion, the present study suggests that signal intensity changes of lung fields during forced breathing using DDR with our previously developed automated tracking system can be an index in assessing pulmonary function in COPD patients. Our findings are important for potentially providing additional insights into COPD pathophysiology, enhancing diagnostic accuracy, and improving patient-centered care. However, further research is needed to validate the utility of DDR in COPD diagnosis and management before widespread clinical adoption.

## Funding Statement

M.N. is supported by 10.13039/100000002NIH (R01CA203636, U01CA209414, R01HL111024, and R01CA240592); H.H. is supported by 10.13039/100000002NIH (R01CA203636, 5U01CA209414, and R01HL135142), NIH/NHLBI (R01HL111024 and R01HL130974); Brigham and Women's Hospital and Tenri Hospital received research funding from Konica-Minolta Inc.

## Ethical statement

This study was approved by the institutional review board of Fukujuji Hospital. Written informed consents were obtained from all the participants.

## CRediT authorship contribution statement

**Noriaki Wada:** Writing – original draft, Visualization, Validation, Methodology, Investigation, Formal analysis, Data curation, Conceptualization. **Akinori Tsunomori:** Software. **Takeshi Kubo:** Writing – review & editing, Visualization, Methodology. **Takuya Hino:** Writing – review & editing, Methodology, Investigation, Data curation, Conceptualization. **Akinori Hata:** Writing – review & editing. **Yoshitake Yamada:** Writing – review & editing. **Masako Ueyama:** Writing – review & editing, Resources. **Mizuki Nishino:** Writing – review & editing. **Atsuko Kurosaki:** Writing – review & editing, Resources. **Kousei Ishigami:** Writing – review & editing. **Shoji Kudoh:** Writing – review & editing, Resources. **Hiroto Hatabu:** Writing – review & editing, Supervision, Project administration, Methodology, Investigation, Funding acquisition, Conceptualization.

## Declaration of Competing Interest

M.N. reports research grants to the institution from Konica Minolta, Inc. for the submitted work; and research grants to the institution from Canon Medical Systems, Inc. and consulting fees from AstraZeneca, outside the submitted work. H.H. reports grants or contracts from Konica Minolta, Inc. for the submitted work; and grants or contracts from Canon Medical Systems, Inc. and consulting fees from Canon Medical Systems, Inc. and Boehringer Ingelheim, outside the submitted work. H.H. also reports patents planned, issued or pending (the provisional US patent application with the serial number 63/610,842). The other authors have no conflicts of interest to disclose related to this article.
