# Supplementary material for: Assessment of pulmonary function in COPD patients using dynamic digital radiography: A novel approach utilizing lung signal intensity changes during forced breathing
Source: Eur J Radiol Open. 2024 Jun 27;13:100579. doi: 10.1016/j.ejro.2024.100579 (PMC11260941; doi:10.1016/j.ejro.2024.100579)
Supplement: Supplementary file 1 — Supplementary material [file mmc1.docx]

**Table S1. Spearman’s correlation coefficients of signal intensity changes (SImax/SImin) of left and right lungs with pulmonary function**

|  | **Left lung** |  | **Right lung** |  |
| --- | --- | --- | --- | --- |
| **Pulmonary function** | **r_s_ (95% CI)** | **P value** | **r_s_ (95% CI)** | **P value** |
| **TV (L)** | 0.18 (-0.04, 0.39) | 0.11 | 0.22 (-0.006, 0.42) | 0.056 |
| **VC (L)** | 0.56 (0.39, 0.70) | <0.0001 | 0.44 (0.25, 0.61) | <0.0001 |
| **%VC (%)** | 0.30 (0.09, 0.49) | 0.0064 | 0.33 (0.12, 0.51) | 0.0029 |
| **FEV_1_ (L)** | 0.44 (0.24, 0.60) | <0.0001 | 0.37 (0.16, 0.55) | 0.0008 |
| **FEV_1_% (%)** | 0.20 (-0.02, 0.40) | 0.077 | 0.17 (-0.05, 0.38) | 0.13 |
| **%FEV_1_ (%)** | 0.19 (-0.03, 0.40) | 0.091 | 0.22 (-0.004, 0.42) | 0.054 |

TV, tidal volume; VC, vital capacity; %VC, percent vital capacity; FEV_1_, forced expiratory volume in one second; FEV_1_%, forced expiratory volume percent in one second divided by forced vital capacity; %FEV_1_, percent predicted FEV_1_, CI, confidence interval.

**Table S2. Multivariate linear regression analysis of signal intensity changes (SImax/SImin) on presence of COPD and confounding factors**

|  | **Estimate** | **Standard error** | **t value** | **P value** |
| --- | --- | --- | --- | --- |
| **COPD** | -0.033 | 0.013 | -2.53 | 0.014 |
| **(Ref: Normal)** |  |  |  |  |
| **Confounding factors** |  |  |  |  |
| **Age** | 0.0006 | 0.001 | 0.56 | 0.58 |
| **Sex, Female** | -0.030 | 0.016 | -1.92 | 0.059 |
| **(Ref: Male)** |  |  |  |  |
| **Height** | 0.003 | 0.002 | 1.72 | 0.089 |

Ref, reference.
